# Local-look navigator gated and cardiac triggered echo-planar spectroscopic imaging of the heart

**DOI:** 10.1186/1532-429X-13-S1-O78

**Published:** 2011-02-02

**Authors:** Kilian Weiss, Nicola Martini, Peter Boesiger, Sebastian Kozerke

**Affiliations:** 1 University and ETH Zurich, Zurich, Switzerland; 2 Fandazione G. Monasterio CNR-Regione Toscana, Massa, Italy

## Introduction

Single-voxel proton magnetic resonance spectroscopy has been shown to be a promising tool for assessing creatine [1] and myocardial triglycerides in humans [2, 3, 4]. A focus of interest is the correlation between triglyceride content in the myocardium and cardiac dysfunction [5]. While spectral information from a single volume is sufficient when alterations with global effects on the heart are studied, a demand for higher and flexible spatial resolution exists when probing local changes. The objective of the current work was to implement a navigated local-look Echo-Planar Spectroscopic Imaging (EPSI) sequence for assessment of triglyceride and creatine content in the myocardium in vivo.

## Methods

**Figure 1:**
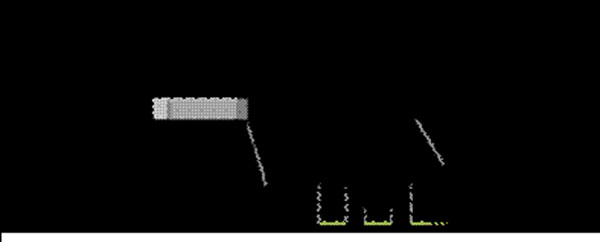
Schematic of the local look navigator gated EPSI sequence a) using echo planar readouts b).

**Figure 2:**
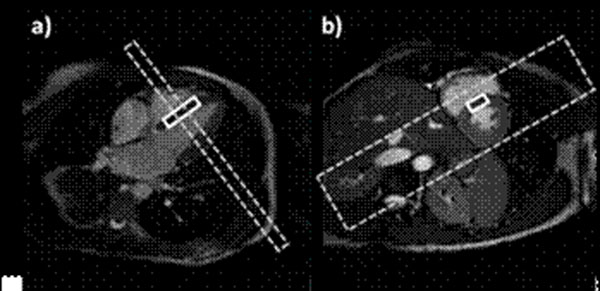
Position of the single voxel (solid line) and the FOX of the EPSI scans (dashed line). (a) Four-chamber view. (b) Short axis view.

Local-look navigator gated spin-echo EPSI (Figure 1) was implemented on a 1.5T Philips Achieva system (Philips Healthcare, Best, The Netherlands). Field-of-excitation (FOX) reduction was based on an optimized selective excitation pulse in phase encoding direction (Figure 2). Pencil-beam navigator echoes were integrated for respiratory gating purposes. Sequence parameters were as follows: echo time: 12 ms, FOV: 300 x 150 mm^2^, FOX: 65 to 85 mm, spatial resolution: 3 x 3 mm^2^, slice thickness: 15 mm, spectral resolution: 4.1 Hz, 8 signal averages, Ernst angle: ~120 deg, cardiac trigger delay: ~320 ms, 5-element cardiac coil array for signal reception. Water-suppressed and unsuppressed EPSI data were acquired in healthy volunteers during free breathing in an average scan time of 2:30 min per average depending on heart rate and respiratory navigator efficiency. For comparison single voxel data were acquired using the navigator gated and cardiac triggered PRESS sequence. The single voxel was placed in the septum to avoid signal contamination from epicardial fat (Figure 2).

## Results

**Figure 3:**
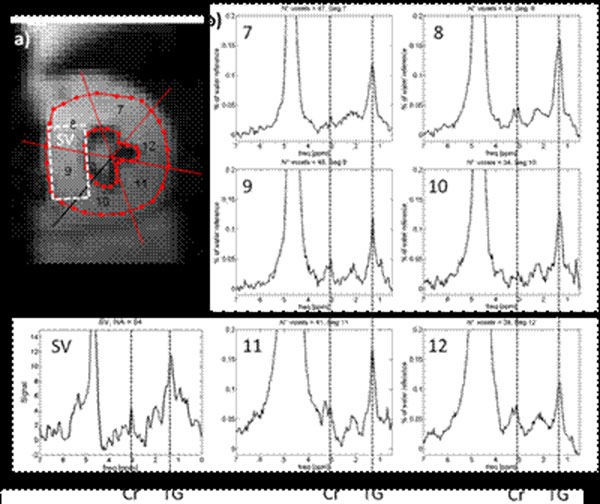
a) Single voxel (SV) and EPCI regions of interst indicated in the EPSI-reference scan. b) Spectra from the regions of interest indicated in a). In all spectra the triglyceride resonance at 1.3 ppm (TG) and the creatine resonance at 3.01 ppm (Cr) can clearly be seen.

EPSI and single voxel spectra are shown in Figure 3. Beside spectra from a region of interest located in the septal wall, spectra from six regions of interest from the mid-cavity are shown for the EPSI scans. In all spectra resonances from triglycerides and creatine are visible.

## Discussion

It has been demonstrated that local look navigator-gated and cardiac triggered spin echo EPSI can be used to assess distributions of myocardial triglyceride and creatine resonances during free-breathing acquisitions. Compared to single voxel techniques the EPSI method provides spectra from different regions of the myocardium. In future work higher B_0_ fields like 3T could be used to increase signal-to-noise ratios, to especially enhance the relatively weak signal of creatine.

